# Survival outcome and prognostic factors in anaplastic oligodendroglioma: a single-institution study of 95 cases

**DOI:** 10.1038/s41598-020-77228-2

**Published:** 2020-11-19

**Authors:** Dong-Won Shin, Seungjoo Lee, Sang Woo Song, Young Hyun Cho, Seok Ho Hong, Jeong Hoon Kim, Ho Sung Kim, Ji Eun Park, Soo Jeong Nam, Young-Hoon Kim

**Affiliations:** 1 Department of Neurological Surgery, Asan Medical Center, University of Ulsan College of Medicine, 88, Olympic-ro 43-gil, Songpa-gu, Seoul, 05505 Republic of Korea; 2 Department of Radiology, Asan Medical Center, University of Ulsan College of Medicine, Seoul, Republic of Korea; 3 Department of Pathology, Asan Medical Center, University of Ulsan College of Medicine, Seoul, Republic of Korea

**Keywords:** Cancer, CNS cancer

## Abstract

The aim of this study was to evaluate prognostic factors including surgical, radiographic, and histopathologic analyses in anaplastic oligodendroglioma (AO) patients. We reviewed the electronic records of 95 patients who underwent surgery and were diagnosed with AO for 20 years. The primary endpoints were progression-free survival (PFS) and overall survival (OS). Univariate and multivariable analyses included clinical, histopathological, and radiographic prognostic factors. Subgroup analysis was performed in isocitrate dehydrogenase (*IDH1/2)*-mutant and 1p/19q-codeleted patients. The median PFS and OS were 24.7 months and 50.8 months, respectively. The 1-, 3-, 5-, and 10-year PFS were 75.8%, 42.9%, 32.4%, and 16.4%, respectively. Furthermore, the 1-, 3-, 5-, and 10-year OS were 98.9%, 76.9%, 42.9%, and 29.7%, respectively. The median PFS and OS of the *IDH1/2*-mutant and 1p/19q-codeleted patients were 54.2 and 57.8 months, respectively. In univariate analyses, young age, frontal lobe, weak enhancement, gross total resection (GTR), low Ki-67 index, 1p/19q codeletion, and *IDH1/2* mutations were associated with a favorable outcome. In multivariable analyses, *IDH1/2* mutation was related to better PFS and OS. In subgroup analysis, GTR was associated with favorable outcomes.

## Introduction

Anaplastic oligodendroglioma (AO) is a rare disease entity, comprising 0.5% of all intracranial neoplasms^1^. The current standard for AO treatment consists of maximum safe resection and radiotherapy (RT) followed by chemotherapy (CTx)^2^.

Since the 2016 World Health Organization (WHO) classification of glioma, molecular studies and chromosomal analysis have been deemed essential for diagnosis. Mutations in the isocitrate dehydrogenase genes (*IDH1/2*) and chromosome 1p/19q codeletion status are key in defining oligodendroglioma. Most previous research usually examined oligodendroglial tumors, which include both oligodendroglioma (OD) and oligoastrocytoma (OA)^3–5^. Hence, research associated with only AO is very rare. Liu et al. reported that 5-year and 10-year survival rate of AO was 50.2% and 36.2%, respectively. They included 1899 patients who had a histological diagnosis only^6^. Kang et al. published a multicenter study of AO which included 376 patients from nine Korean institutes. They reported that 5-year and 10-year survival rate of AO was 58% and 45%, respectively. The median PFS was 34.5 months, and the actuarial 5- and 10-years PFS rates were 38% and 24%, respectively^7^. The limitation of those studies was histological diagnosis, not molecular diagnosis. Our study tries to overcome this limitation by subgroup analysis which included molecular diagnosis as *IDH1/2*-mutant and 1p/19q codeleted AO.

The aim of this study was to evaluate prognostic variables, including extent of surgical resection (EOR), radiographic features, and molecular markers, in a large single-center series of AO patients. In addition, according to these results, we performed a subgroup analysis in *IDH1/2*-mutant and 1p/19q-codeleted AO patients to predict their prognosis.

## Materials and methods

### Patient eligibility

We reviewed the electronic records of patients who underwent surgical procedures at our institute and were diagnosed with AO from January 1998 to December 2018.

Eligibility criteria.

1. Underwent surgery for the first time for anaplastic oligodendroglioma
2. Age at surgical procedure ≥ 20 years old
3. Followed for more than 1 year after surgery
4. EOR evaluated by pre- and postoperative MRI
5. No duplicated or recurrent cases

### Study design

The primary endpoints of this study were progression-free survival (PFS) and overall survival (OS). The date of diagnosis was defined as the date of initial surgery and used as the starting date for PFS and OS, with survival measured until either tumor growth was noted on imaging follow-up, latest follow-up, or death.

We performed clinical analyses in 95 AO patients to determine predictive factors for PFS and OS. Subsequently, subgroup analysis was done for patients who had both *IDH1/2*-mutant and 1p/19q codeletion, which is the current molecular definition of AO.

### Extent of resection and adjuvant treatment

EOR was assessed by comparing preoperative CT and MRI with postoperative CT and MRI, which was usually taken in the 48 h following surgery. Gross total resection (GTR) was defined as the removal of more than 99% of enhancing lesions^8^.

Adjuvant therapy comprises RT, CTx in the form of either PCV (procarbazine, lomustine, and vincristine) or temozolomide, or RT followed by CTx. Chemotherapeutic regimens followed the standard of treatment^9^. RT was performed with three-dimensional (3D) conformal RT or intensity-modulated RT. The RT dose was 60 Gy over 30 fractions in most cases. The RT volume was the surgical bed and T2 (or FLAIR) hyperintensity on postoperative MRI plus a margin of 1.5–2 cm.

### Radiographic evaluation

**Figure 1:**
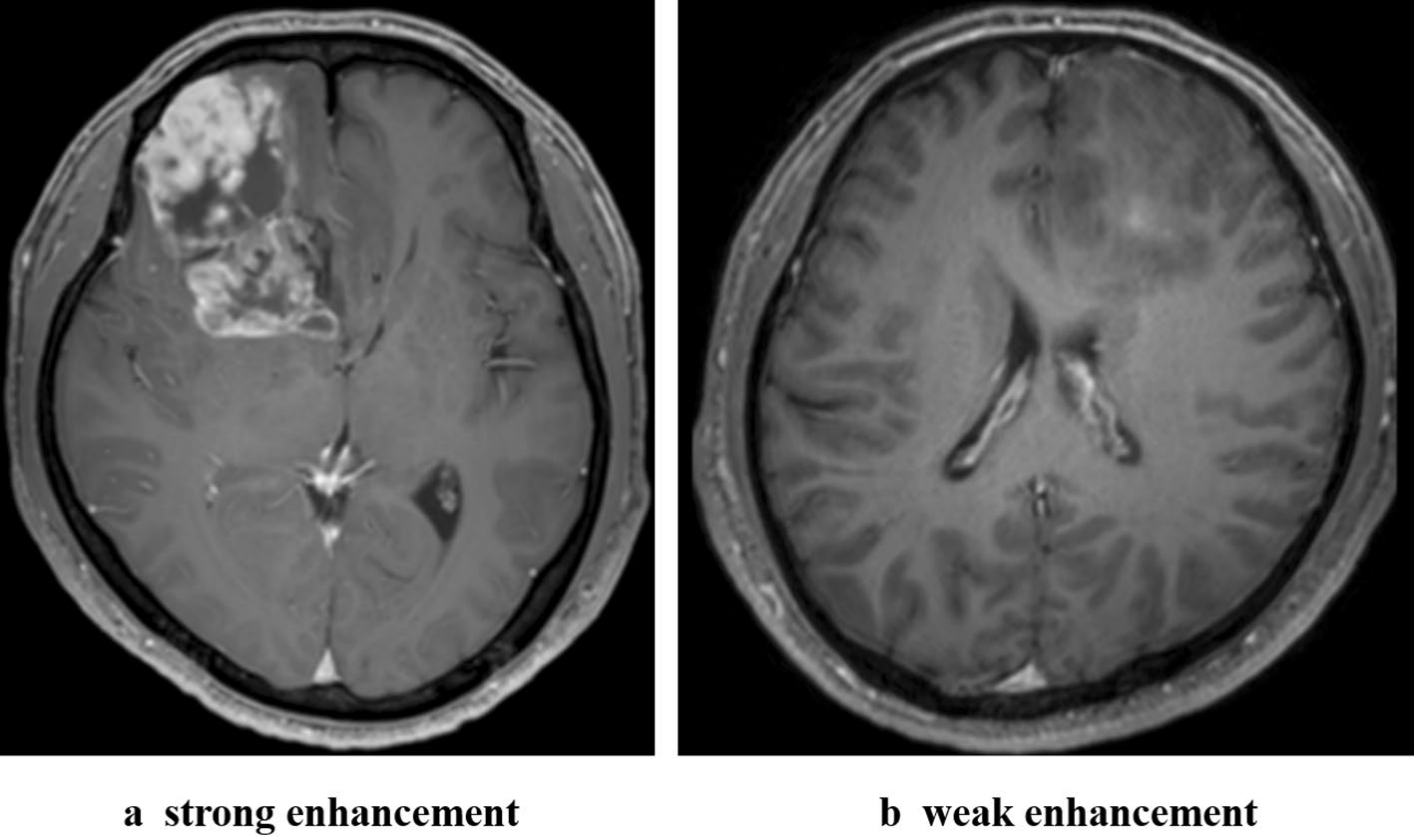
Classification of enhancement pattern according to T1 gadolinium-enhanced MRI.

Periventricular lesions (ie, subventricular zone involvement) were defined as any T2-weighted, FLAIR, or T1-Gd-enhanced portion contacting any ventricular system. Calcification was assessed according to preoperative CT. Enhancement patterns were classified into two categories: strong or weak (Fig. 1). Strong enhancement was defined by rim enhancement, whereas weak enhancement was patchy, focal, and faint enhancement. Tumor progression was assessed on follow-up MRI according to The Response Assessment in Neuro-Oncology (RANO) criteria for gliomas^10^. When in doubt, our standard of practice was to use MR spectroscopy and positron emission tomography.

### Histopathologic factors

The Ki-67 labeling index was defined as the percentage of cells with positive nuclear stain. The fraction of labeled nuclei was determined by eye using a grid and expressed as a percentage of five intervals. Oligodendroglial tumors have been routinely tested for *IDH*1 mutation status using immunohistochemistry (*IDH*1 R132H, MPQ-67, 1:50 dilution; Cell Marque, Rocklin, CA) since 2012 in our institute. Subsequently, DNA sequencing was performed for negative *IDH*1 immunohistochemistry. Exon 4 of the *IDH1* and *IDH2* genes was amplified by polymerase chain reaction and sequenced from tumor samples, as done by Yan et al.^11^ 1p/19q codeletion status was determined by fluorescence in situ hybridization (FISH). This test was performed using Vysis (Abbott Laboratories, Chicago, IL) 1p36/1q25 and 19q13/19p13 FISH probes. At least 60 tumor cells were counted, and a specimen was considered positive if ≥ 30% of tumor cells demonstrated patterns corresponding to 1p/19q deletion.

### Statistical analysis

Data analysis was performed using SPSS Statistics for Windows (version 21.0; IBM, Armonk, NY) and GraphPad Prism 8 (GraphPad Software, San Diego, CA). A significance level of *p* < 0.05 (*) denoted statistical significance. Univariate analyses were performed with the log-rank test. For multivariable analysis, the Cox proportional hazards method was performed to obtain independent predictors for PFS and OS of AO patients. Among the variables included in the univariate analyses, those with *p* < 0.05 were selected for multivariable analyses. The prognostic factors were analyzed using the logistic regression model with a backward stepwise method.

### Ethics approval

All procedures performed in studies involving human participants were in accordance with the ethical standards of the institutional research committee (Asan Medical Center, Reference No. 2020-0738) and with the 1964 Helsinki declaration and its later amendments or comparable ethical standards.

### Consent to participate

Because of its retrospective manner, informed consent was waived.

## Results

### Patient and tumor characteristics

We identified 191 cases in total from January 1, 1998 to December 31, 2018 at our institute. According to our eligibility criteria, 96 cases were excluded. The excluded cases included 62 for follow-up less than 1 year, 14 for not clear pathologic confirm, 9 for duplication, 5 for unavailable preoperative MRI, 4 for age under 20, and 2 for other reasons. Finally, 95 patients were included in the present study. Among them, 31 patients were *IDH1/2*-mutant and 1p/19 codeletion AO, whereas other 64 patients were only histologically proven AO without molecular analysis, which was defined with AO, not otherwise specific (NOS).

**Figure 2:**
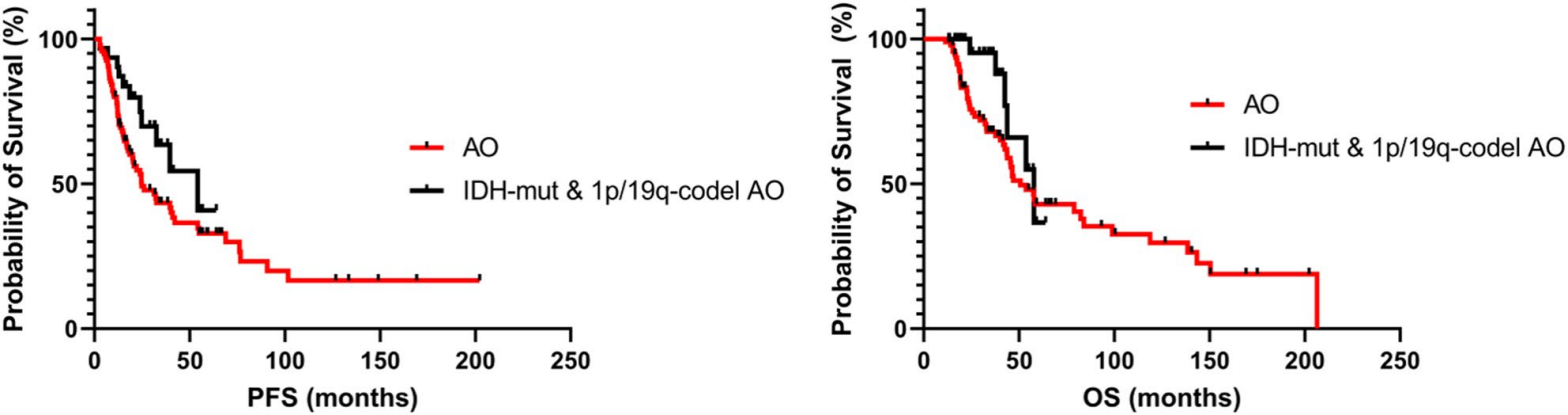
Kaplan–Meier survival curve for all anaplastic oligodendroglioma (AO) patients and *IDH1/2*-mutant and 1p/19q codeletion AO patients.

A total of 59 males and 36 females with a mean age of 50 years (range: 24–75) were included. The median PFS was 19.0 months (range: 2.8–202.3 months) and the median OS was 37.7 months (range: 12.0–202.3 months). The progression-free 1-, 3-, 5-, and 10-year survival was 75.8%, 42.9%, 32.4%, and 16.4%, respectively. Furthermore, the overall 1-, 3-, 5-, and 10-year survival was 98.9%, 76.9%, 42.9%, and 29.7%, respectively (Fig. 2).

Table 1 includes characteristics and epidemiological data of our study population. The predominant tumor location was the frontal lobe in 67 patients (70.5%) followed by multiple lobes in 12 patients (12.6%). Kaplan–Meier analysis revealed a frontal lobe location to be a favorable prognostic factor for PFS (*p* = 0.021, HR 0.36, 95% CI 0.17–0.74). Age under 60 was favorable prognostic factor for OS (log-rank test, median OS 78.9 vs. 32.9 months, HR 0.22, *p* = 0.0002).

**Table 1:** Characteristics of patients with anaplastic oligodendroglioma (AO) and the *IDH1/2*-mutant and 1p/19q-codeleted AO subgroup.

Anaplastic oligodendroglioma		*IDH*1/2-mutant, 1p19q-codeleted AO subgroup	
Variable	Results (n = 95)	Variable	Results (n = 31)
Age (mean; range)	50 (24–75)	Age (mean; range)	51 (24–75)
Median follow up period (range)	34.1 months (11.9–202.3)	Median follow up period (range)	32.2 months (13.1–64.0)
Median survival duration	50.8 months	Median survival duration	57.8 months
Sex (%)	Male : 59 (62)/Female : 36 (37)	Sex (%)	Male : 16 (51) /Female : 15 (48)
**Location (%)**		**Location (%)**	
Frontal	67 (70)	Frontal	28 (90)
Temporal	10 (10)	Temporal	1 (3)
Occipital	4 (4)	Occipital	0
Parietal	2 (2)	Parietal	0
≥ 2 lobes	12 (12)	≥ 2 lobes	2 (6)
**Extent of resection (%)**		**Extent of resection (%)**	
GTR	43 (45)	GTR	9 (29)
Non-GTR	45 (47)	Non-GTR	22 (70)
Bx	7 (7)		
**Type of treatment (%)**		**Type of treatment (%)**	
Surgery or Bx only	6 (6)	Surgery or Bx only	1 (3)
Surgery + RT	36 (37)	Surgery + RT	6 (19)
Surgery + CTx	5 (5)	Surgery + CTx	4 (12)
Surgery + RT + PCV	21 (22)	Surgery + RT + PCV	7 (22)
Surgery + RT + Temodal	27 (28)	Surgery + RT + Temodal	13 (41)
**Ki-67 index (median = 20**%**)**		**Ki-67 index**	
≥ 20%	39 (54)	≥ 20%	9 (39)
< 20%	33 (45)	< 20%	14 (60)
**Enhancement pattern (%)**		**Enhancement pattern (%)**	
Strong	61 (64)	Strong	16 (51)
**Calcification (%)**		**Calcification (%)**	
Yes	34 (36)	Yes	19 (61)
**Periventricular involvement (%)**		**Periventricular involvement (%)**	
Yes	76 (80)	Yes	24 (77)
***IDH1/2-mutant status (%)***			
*IDH1/2*-mutant	32 (33)		
*IDH1/2*-wildtype	12 (12)		
Unavailable	51 (53)		
**1p/19q codeletion status (%)**			
Codeleted	54 (56)		
No	18 (18)		
Unavailable	23 (24)		

*AO anaplastic oligodendroglioma; *IDH* Isocitrate dehydrogenase; *GTR* gross total resection; *Bx* biopsy; *CTx* chemotherapy; *RT* radiotherapy; *PCV* procarbazine + lomustine + vincristine.

### Extent of resection and adjuvant treatment

GTR was achieved in 43 of 88 (48.9%) patients. In univariate analysis (log-rank test), the GTR group had a significantly longer PFS than non-GTR groups (median PFS 41.1 vs. 23.9 months, HR 0.58, 95% CI 0.35–0.97, *p* = 0.038). However, there was no significant difference in OS (median OS 57.8 vs. 47.0 months, *p* = 0.148).

Thirty-six (37.8%) patients received adjuvant RT without CTx whereas five (5.2%) patients were given CTx without RT. Among 36 patients who received RT but not CTx, they were included in KNOG-1101 study for RT single arm or had co-morbidities. Forty-eight (50.5%) patients had RT followed by CTx. Specifically, 27 of 48 (56.3%) patients had temozolomide and 21 (43.8%) had PCV chemotherapy. Chemotherapy regimens were decided by patients themselves based on socioeconomic status, comorbidities, possibilities of adverse effects, and compliance. Univariate analysis (log-rank test) based on adjuvant treatment methods showed that the adjuvant RT/PCV group had a better PFS than those with adjuvant RT followed by temozolomide (median PFS 32.2 vs. 24.7), but this was not significant (*p* = 0.621). The median OS for each group was 57.6 months and 57.8 months, respectively (*p* = 0.573).

### Radiographic evaluation

Ninety-three patients had preoperative CT scans. Among them, 59 patients (63.4%) had no calcification on their CT scan. Seventy-six (80.0%) patients had periventricular involvement. The log-rank test revealed that calcification was not associated with PFS or OS as well as periventricular involvement.

Enhancement patterns varied among AO patients. Sixty (63.1%) patients had strong or rim enhancement, whereas 35 had weak or no contrast enhancement. Strong enhancement was a negative prognostic factor for PFS and OS (median PFS 18.6 vs. 42.1 months, HR 1.98, *p* = 0.014; median OS 45.5 vs. 84.1 months, HR 1.68, *p* = 0.094).

### Histologic and molecular analysis

Ki-67 index data was available in 72 patients, with a median Ki-67 index of 20%. Thirty-nine (54.1%) patients had a Ki-67 index of more than 20%. In univariate analysis (log-rank test), those with Ki-67 < 20% showed a significantly longer PFS and OS (median PFS 54.9 vs. 16.9 months, HR 0.31, *p* = 0.029; median OS 82.4 vs. 45.7 months, HR 0.46, *p* = 0.010).

*IDH1* or *IDH2* mutation status was available in 44 patients. Among them, 32 patients had *IDH1/2* mutations whereas 12 patients did not. The *IDH1/2*-mutant group also had a significantly longer PFS and OS than the *IDH1/2*-wildtype group (median PFS 54.2 vs. 9.5 months, HR 0.1753, *p* < 0.0001; median OS 57.8 vs. 22.8 months, HR 0.07929, *p* < 0.0001).

### Multivariable analysis

**Figure 3:**
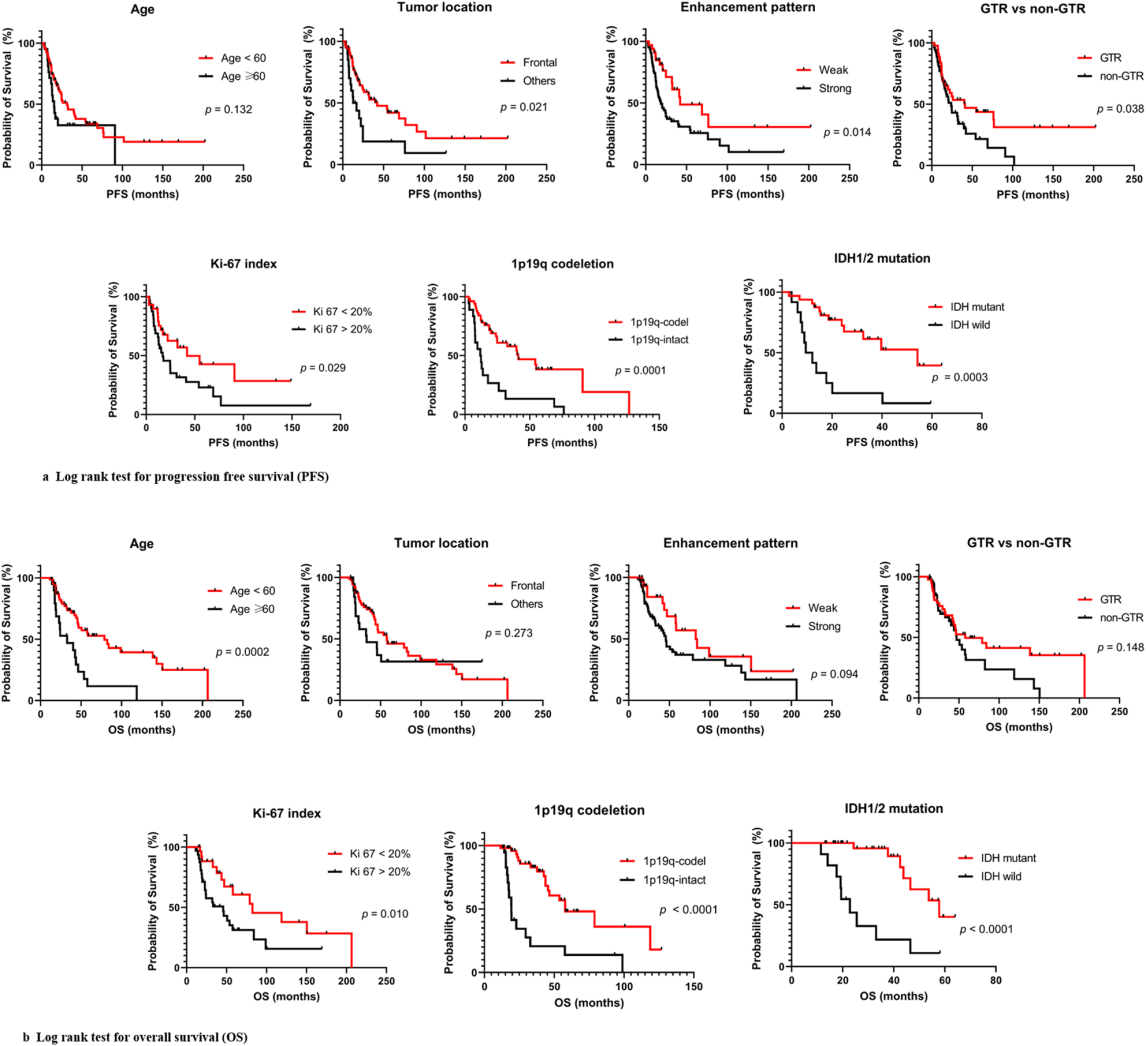
Univariate analysis (log-rank test) for (**a**) progression-free survival (PFS) and (**b**) overall survival (OS).

Figure 3 and Table 2 show the results of univariate and multivariate analyses in AO patients. Because of the limited sample size, we could not include all variables found to be of prognostic significance in the univariate analyses in a multivariable Cox model. The variables included in multivariable analyses for PFS were frontal lobe location, weak enhancement, GTR, lower Ki-67 index, 1p/19q codeletion, and *IDH1/2* mutation. Of these, *IDH1/2* mutation remained significant for PFS (HR 0.284, *p* = 0.018). Furthermore, we performed multivariable analyses for OS. Included variables were age under 60, lower Ki-67 index, 1p/19q codeletion, and *IDH1/2* mutation. Among them, Ki-67 < 20% (HR 0.16, *p* = 0.034) and *IDH1/2* mutation (HR 0.12, *p* = 0.004) were significant favorable prognostic factors for OS.

**Table 2:** Favorable prognostic factors of progression-free and overall survival in patients with anaplastic oligodendroglioma.

Factors	Progression-free survival (n = 95)				Overall survival (n = 95)			
	Univariate		Multivariate (backward stepwise)		Univariate		Multivariate (backward stepwise)	
	*p* value	HR (95% CI)	*p* value	HR (95% CI)	*p* value	HR (95% CI)	*p* value	HR (95% CI)
Sex (female)	0.836	0.95 (0.56–1.60)			0.780	0.92 (0.51–1.66)		
Age (< 60 years)	0.132	0.64 (0.35–1.15)			< 0.001*	0.37 (0.19–0.61)	0.703	1.02 (0.93–1.11)
Frontal lobe tumor	0.021*	0.52 (0.29–0.91)	0.919	0.92 (0.18–4.60)	0.273	0.70 (0.38–1.32)		
No strong enhancement	0.014*	0.49 (0.27–0.88)	0.876	0.90 (0.25–3.27)	0.094	0.59 (0.31–1.10)		
Calcification	0.448	0.81 (0.46–1.41)			0.083	0.57 (0.30–1.09)		
No periventricular involvement	0.417	0.75 (0.37–1.52)			0.459	0.76 (0.37–1.57)		
Gross total resection	0.038*	0.57 (0.37–0.98)	0.560	0.71 (0.22–2.24)	0.148	0.66 (0.37–1.16)		
Methylated *MGMT* promoter	0.089	0.29 (0.63–1.32)			0.093	0.18 (0.02–1.72)		
Adjuvant treatment	0.695	0.77 (0.47–1.28)			0.385	0.74 (0.42–1.30)		
Ki-67 index < 20%	0.029*	0.52 (0.28–0.94)	0.099	0.40 (0.14–1.18)	0.010*	0.42 (0.21–0.83)	0.034*	0.16 (0.28–0.87)
1p/19q codeletion	< 0.001*	0.30 (1.59–0.57)	0.982	0.98 (0.16–5.91)	< 0.001*	0.22 (0.11–0.45)	0.234	4.06 (0.36–45.43)
*IDH1/2* mutation	< 0.001*	0.25 (0.11–0.56)	0.018*	0.28 (0.10–0.80)	0.004*	0.26 (0.01–0.70)	0.004*	0.12 (0.03–0.51)

*HR* hazards ratio; *CI* confidence interval; *MGMT* O-6-methylguanine-DNA methyltransferase.

*p < 0.05.

### *IDH1/2*-mutant and 1p/19q codeletion AO group analysis

Thirty-one patients were eligible for the 2016 WHO classification of *IDH1/2*-mutant and 1p/19q codeletion AO. The mean age in this group was 51 years (24–75 years) and the median follow-up period was 34.1 months (13.1–64.0 months). Sixteen males and 15 females were included. Disease progression was observed in 11 patients (35.5%) and 6 (19.3%) died during the follow-up period. The median PFS and OS were 54.2 months and 57.8 months, respectively. Progression-free 1-, 3-, and 5-year survival rates were 90.3%, 63.5%, and 40.8%, respectively. Furthermore, overall 3- and 5-year survival rates were 87.9% and 36.6%, respectively. Univariate analysis was performed based on age, EOR, radiographic findings; enhancement pattern, calcification, or periventricular involvement, and Ki-67 index (< 20% vs. ≥ 20%). Among these factors, the GTR group had a significantly longer PFS than the non-GTR group (HR 0.25, 95% CI 0.07–0.88, *p* = 0.030), but there was no significant difference in OS.

## Discussion

### Extent of resection and adjuvant therapy

According to the National Comprehensive Cancer Network (NCCN) 2019 guideline, anaplastic glioma was recommended to be treated by maximal safe resection followed by RT and adjuvant PCV (category 1)^12,13^. Shin et al. reported that adjuvant chemotherapy was an independent prognostic factor for improved OS^14^. Numerous studies highlight EOR in association with prognosis^2,6–8,15–17^. Based on our results, we should attempt GTR which could be enhanced by 5-aminolevulinic acid (5-ALA)^16^.

PCV chemotherapy has been the standard chemotherapeutic regimen for grade 2 or 3 glioma patients^14,18^. In contrast, temozolomide has been utilized as an adjuvant treatment for AO patients^18^. Our study revealed that RT followed by PCV chemotherapy yielded a longer median PFS (32.3 vs. 24.7 months, *p* = 0.6209) than RT with temozolomide; however, OS was not significantly different between these groups (57.6 vs. 57.8 months, *p* = 0.5729). PCV and temozolomide have their own advantages and disadvantages for patients. Our results support the notion that temozolomide is not inferior to PCV in relation to OS, although this is cannot be concluded with certainty as this was a retrospective study.

### Radiographic evaluation

It is difficult to differentiate AO from OD (WHO grade II) based on CT or MRI, as OD and AO share radiographic findings including calcification and enhancement. There are slight but unspecific differences in MRI. OD also shows minimal or moderate enhancement in many cases^19^. However, strong enhancement or rim enhancement patterns are rare. Additionally, the incidence of calcification in our AO patients was relatively lower than in OD patients (90% previously reported^19^ vs. 36% in our population).

Prognosis related to enhancement pattern has been evaluated in anaplastic astrocytomas in several studies^20^. However, this is the first study to examine prognosis and enhancement patterns of only AO, although our results did not reach statistical significance in multivariable analysis. Based on our study, we predict relatively earlier disease progression after surgery in patients with strong enhancement.

Periventricular involvement has been regarded as a poor prognostic factor in GBM patients^21^. Lim et al. revealed that a GBM involving neural stem cell regions (like the subventricular zone) tends to have multifocal lesions and a greater rate of recurrence, resulting in poorer outcomes. However, in the present study, we could not find a relationship between periventricular involvement and survival outcome.

### Ki-67 index

The Ki-67 index has been evaluated in several studies related to AO. Preusser et al. reported that a low Ki-67 index was associated with significantly longer PFS and OS in univariate analysis^4^. Furthermore, Celso et al. revealed that high Ki-67 correlated with shorter OS, both in univariate and multivariable analyses^22^. In our study, a lower Ki-67 index was related to a better survival outcome (HR 0.16, 95% CI 0.28–0.87, *p* = 0.034).

### IDH1/2-mutant and 1p/19q-codeleted AO patients

1p/19q codeletion and *IDH1/2* mutation are well-known favorable factors in OD patients^23^. After the introduction of the 2016 WHO classification, *IDH1/2* mutation status was key for classifying glioma patients. In our institute, *IDH1/2* mutation status of glioma patients has been routinely available since June 2012. Other patients without data on *IDH1/2* mutation or with *IDH1/2*-wild type AO were diagnosed with AO, NOS. Clinically, there are many AO patients who have been followed up without molecular analysis results. To include patients with unknown *IDH1/2* mutation status but diagnosed with AO before the 2016 WHO classification, our study included all AO patients before and after 2016. We tried to identify predictive factors in AO patients. Subsequently, we were expecting to reach a similar conclusion in *IDH1/2*-mutant and 1p/19q-codeleted AO patients. Fortunately, among the variables which were meaningful in AO patients, GTR was associated with PFS in univariate analysis for *IDH1/2*-mutant and 1p/19q-codeleted AO patients. Due to the small number of patients, we were unable to make a strong conclusion in our study. However, this result can give direction for further study. Further larger studies (or meta-analyses) should consider the factors mentioned in this study (enhancement pattern, Ki-67 index, and EOR) and determine their exact prognostic value.

## Conclusion

Age under 60 and lower Ki-67 index were associated with longer OS. In subgroup analysis of *IDH1/2*-mutant and 1p/19-codeleted AO patients, the GTR group had better PFS than the non-GTR group. We recommend further studies including our factors to determine associations between PFS or OS in *IDH1/2*-mutant and 1p/19q-codeleted AO patients.
